# Editorial: Ethnofood chemistry: bioactive components in unexploited foods from centres of biodiversity

**DOI:** 10.3389/fnut.2023.1232223

**Published:** 2023-07-20

**Authors:** Yasmina Sultanbawa, Dejian Huang, Michael Rychlik

**Affiliations:** ^1^Centre for Nutrition and Food Sciences, ARC Training Centre for Uniquely Australian Foods, Queensland Alliance for Agriculture and Food Innovation, The University of Queensland, Brisbane, QLD, Australia; ^2^Department of Food Science and Technology, National University of Singapore, Singapore, Singapore; ^3^Analytical Food Chemistry, Department of Life Science Engineering, School of Life Sciences, Technical University of Munich, Freising, Germany

**Keywords:** traditional foods, nutrients, health, bioactive properties, safety

Billions of people globally lack access to a healthy diet which is a leading cause of many noncommunicable diseases and individual foods and nutrients are important for diet diversity which is critical for maintaining health. Dietary patterns of different populations are based on a combination of different foods and there is an interaction in relation to health between foods and nutrients when consumed together. This is where ethnofoods (traditional foods) as unexploited and underutilized food can make a significant change to health and wellbeing by contributing to the diversity of foods needed in the diet.

Ethnofoods are consumed by Indigenous populations worldwide and form an integral component of the unexploited foods from the Centers of Biodiversity. They are nutrient-dense and are rich sources of micro nutrients. In addition, due to the occurrence of vitamins, trace elements, antioxidants and important dietary phytochemicals, diet diversification with ethno-plant foods is a sustainable and affordable strategy for health promotion of these populations and offers dietary perspectives also for other regions in the world.

The aim of this Research Topic is to feature the importance of incorporating ethno plant foods in the diet not only to provide nutrition and health but also to the role these plants played in different centers of biodiversity. This Research Topic is focussed on looking at ethno plant foods from Centers of Biodiversity (Africa, Asia and Australia, Central America, South America) with bioactive components of nutritional and health value.

There is a total of 10 manuscripts included in this Research Topic. Two studies explored the physicochemical and bioactive characterization and *in vitro* health properties of the Peruvian Andean maize (*Zea mays* L.). Fuentes-Cardenas et al. studied the Peruvian Andean maize race *Cabanita* with respect to its bioactive profiles, physical characteristics, and *in vitro* antioxidant properties. The diversity of the Peruvian Andean maize race *Cabani*ta from two provinces (Caylloma and Castilla) in the Arequipa region showed that it was a promising source of phenolic compounds (p-coumaric and ferulic acid derivatives). The anthocyanins were only present in samples that were partially red and contained purple-pigmented kernels. Ranilla et al. investigated the variability of the metabolites in three maize types (white, red and orange) from the same Peruvian Andean race *Cabanita*. Maize with different pigmentation from the *Cabanita* race showed variations in primary and secondary metabolite composition (phenolic and carotenoid) and was different at different stages of grain maturity. Most phenolic compounds decreased with kernel maturity in all *Cabanita* maize types. All maize types showed similar fatty acid contents which increased with kernel development. In general, all *Cabanita* maize types had similar *in vitro* health-relevant functional properties which were reduced significantly with grain development. Another two studies presented work on Australian native plant foods that have been consumed by the Indigenous population for thousands of years. A recent study on charred plant food remains from Madjedbebe rockshelter in northern Australia, dated the use of these plants back to between 65,000 and 53,000 years. Fyfe, Hong et al. reported on the folate vitamers of the green plum, a native fruit of Australia that grows on the tree *Buchanania obovata* in the northern parts of the Northern Territory and Western Australia. This study revealed that green plums are a good natural dietary source of folates. The green plum 5-methyltetrahydrofolate form of folate increases and accumulates in the fruit through development, ripening and senescence. Akter et al. studied the *in vitro* bioaccessibility and intestinal absorption of selected bioactive compounds in *Terminalia ferdinandiana* (or Kakadu plum), a native Australian fruit. The intestinal absorption assay revealed that the phenolic compound ellagic acid, had the highest permeability *in vitro*. For ascorbic acid, even though bioaccessibility was high, *in vitro* permeability was low, indicating that different compounds show variation in bioaccessibility and absorption.

Grosshagauer et al. studied the potential of Moringa foods from a food chemistry perspective. *Moringa oleifera* is native to India but is also prevalent in tropical and subtropical regions of Africa, Asia, Central and South America. This mini-review looked at the food chemistry and nutrition-related data on Moringa preparations. The dietary intake of the leaves of *Moringa oleifera* is high and has been considered safe, even though variations in different processing and extraction methods prevent the comparison of data and also affect the formulation of guidelines. High levels of anti-nutrients and contaminants indicate the need for further studies, and this should inform legal regulations for the dietary intake of *Moringa oleifera* products. Another study by Chen et al. shows the incorporation of the extract of *Psychotria viridiflora* stem (an antidiabetic tropical plant found in Sarawak, East Malaysia) in noodles and the effect on α-amylase and α-glucosidase. *P. viridiflora* α-amylase inhibition activity is higher than α-glucosidase inhibition activity. In general, the fortification of *P. viridiflora* to develop functional noodles could provide a healthier alternative to diabetic patients. The next three studies are reporting on the therapeutic properties of ethnoplants used in different applications. Yang et al. reported on Rosa roxburghii Tratt (*R. roxburghii*) tea, a traditional Chinese beverage. Two cultivars of *R. roxburghii* were compared for their phenolics and bioactive properties. Both cultivars showed good phenolic contents and potent antioxidant capacity, therefore the leaves could be a promising source of phenolic compounds for functional beverages. Pathaw et al. did a comparative review of the anti-nutritional factors of herbal tea concoctions. This review focussed on underutilized plants of North-East India, *Cymbopogon citratus* (Lemongrass), *Rhus chinensis* (Heimang), *Nelumbo nucifera* (Lotus), *Mentha spicata* (Spearmint) and others, used as herbal tea and processing methods to understand the reduction in anti-nutrients. Different processing methods such as drying or heating process help remove anti-nutrients, and future research is required on adapting brewing conditions to optimize anti-nutrient reduction. Sultana et al. reported on assessing extracts of *Dillenia pentagyna* Roxb. for pharmacological potential and related to the traditional use of this plant to treat cancer, wound healing, and diabetes by local tribes in Bangladesh. Finally, Fyfe, Smyth et al. presented a food chemists perspective paper on developing a framework for responsible research for Australian native plant foods, most of which are ethnofoods eaten by Australia's Indigenous people. This perspective highlights the importance for food chemists to uphold the rights of Indigenous Australians when working with communities growing the food on their land and complying with both local and international laws and obtaining permits and benefit-sharing agreements where required.

## Author contributions

All authors listed have made a substantial, direct, and intellectual contribution to the work and approved it for publication.

